# Substance use and symptoms of mental health disorders: a prospective cohort of patients with severe substance use disorders in Norway

**DOI:** 10.1186/s13011-021-00354-1

**Published:** 2021-02-27

**Authors:** Christer Frode Aas, Jørn Henrik Vold, Rolf Gjestad, Svetlana Skurtveit, Aaron Guanliang Lim, Kristian Varden Gjerde, Else-Marie Løberg, Kjell Arne Johansson, Lars Thore Fadnes, for the INTRO-HCV Study Group, Vibeke Bråthen Buljovcic, Fatemeh Chalabianloo, Jan Tore Daltveit, Silvia Eiken Alpers, Trude Fondenes Eriksen, Per Gundersen, Velinda Hille, Kristin Holmelid Håberg, Rafael Alexander Leiva, Siv-Elin Leirvåg Carlsen, Martine Lepsøy Bonnier, Lennart Lorås, Mette Hegland Nordbotn, Cathrine Nygård, Maria Olsvold, Christian Ohldieck, Lillian Sivertsen, Hugo Torjussen, Jan-Magnus Økland, Tone Lise Eielsen, Nancy Laura Ortega Maldonado, Ewa Joanna Wilk, Ronny Bjørnestad, Ole Jørgen Lygren, Marianne Cook Pierron, Olav Dalgard, Håvard Midgard, Peter Vickerman

**Affiliations:** 1 Bergen Addiction Research group, Department of Addiction Medicine, Haukeland University Hospital, Bergen, Norway; 2 Department of Global Public Health and Primary Care, University of Bergen, Bergen, Norway; 3 Division of Psychiatry, Haukeland University Hospital, Bergen, Norway; 4 Norwegian Centre for Addiction Research, University of Oslo, Oslo, Norway; 5 Department of Mental Disorders, Norwegian Institute of Public Health, Oslo, Norway; 6 Population Health Sciences, Bristol Medical School, University of Bristol, Bristol, UK; 7 Department of Clinical Psychology, University of Bergen, Bergen, Norway

**Keywords:** Substance use disorder, Substance abuse, Mental disorder, Psychological distress, Mental health problems, Opioid substitution treatment, Opioid dependence

## Abstract

**Background:**

There is high co-occurrence of substance use disorders (SUD) and mental health disorders. We aimed to assess impact of substance use patterns and sociodemographic factors on mental health distress using the ten-item Hopkins Symptom Checklist (SCL-10) over time.

**Methods:**

Nested prospective cohort study of 707 participants with severe SUD across nine opioid-agonist-therapy outpatient clinics and low-threshold municipality clinics in Norway, during 2017–2020. Descriptive statistics were derived at baseline and reported by means and standard deviation (SD). A linear mixed model analysis was used to assess the impact of substance use patterns and sociodemographic factors on SCL-10 sum score with beta coefficients with 95% confidence intervals (CI).

**Results:**

Mean (SD) SCL-10 score was 2.2 (0.8) at baseline with large variations across patients. We observed more symptoms of mental health disorders among people with frequent use of benzodiazepines (beta 3.6, CI:2.4;4.8), cannabis (1.3, CI:0.2;2.5), opioids (2.7, CI:1.1;4.2), and less symptoms among people using frequent stimulant use (− 2.7, CI:-4.1;-1.4) compared to no or less frequent use. Females (1.8, CI:0.7;3.0) and participants with debt worries (2.2, CI:1.1;3.3) and unstable living conditions (1.7, CI:0.0;3.3) had also higher burden of mental health symptoms. There were large individual variations in SCL-10 score from baseline to follow-up, but no consistent time trends indicating change over time for the whole group. 65% of the cohort had a mean score > 1.85, the standard reference score.

**Conclusions:**

People with SUD have a considerable burden of mental health symptoms. We found no association between substance use patterns and change in mental health symptoms over time. This could suggest that the differences observed were indicating flattening of effects or self-medication to a larger degree than medication-related decline in mental health. This call for better individualized mental health assessment and patient care.

**Supplementary Information:**

The online version contains supplementary material available at 10.1186/s13011-021-00354-1.

## Background

Substance use disorders (SUD) contribute to 11.8 million deaths globally per year and 1.5% of the global disease burden [1]. It is estimated that 2% of the world population has a SUD, with some countries reporting a prevalence of SUD greater than 5% [1]. More than half of the people with a SUD will experience a mental health disorder at some point during their lives [2, 3], yet it is less clear whether mental health disorders develop mostly as a consequence of substance use or vice versa [4]. The co-occurrence of SUD and mental health disorders may be attributed to shared genetic vulnerability and pathophysiological processes possibly related to specific neurotransmitter systems [5, 6]. Even though most research has been in relation to amphetamines, cannabis and alcohol, comorbid mental health symptoms are probably also the case for the more severe forms of SUD like opioid dependence. However, less is known about the prevalence, predictors and change over time of mental health symptoms in these patient groups, limiting optimal clinical care. It has been suggested that these comorbidities often are under-recognized in clinical settings [7, 8].

Among people with SUD in Europe, the most prevalent mental health disorders in epidemiological studies are personality disorders (51%), mood disorders (35%), attention-deficit hyperactivity disorder (30%) and anxiety disorders (27%) [9–12]. Poor quality of life [13], concurrent drug use, including benzodiazepine misuse (e.g. without prescription, higher frequency or dosage than prescribed), is common and prevalent among SUD and people enrolled in opioid agonist therapy (OAT) [14, 15]. Some research suggest that benzodiazepine misuse are associated with other substance use, aggressive behavior and worsening mental health symptoms and disorders [16, 17]. Having a SUD, or a mental health disorder, is also likely to increase the risk for misuse of opioids [18, 19]. Opioid dependence is the most severe SUD, and of all illegal drugs, opioids represents the most fatal risk factor, the highest disease burden and most urgent demand for treatment [20, 21]. In addition, substance use patterns of cannabis and simulants especially frequent use, are found to be associated with residual cognitive impairment and poor mental health [22–24].

Attention to mental health symptoms could perhaps better facilitate and optimize individualized mental health care and SUD treatment to these marginalized and vulnerable populations in low-threshold settings and OAT programs. It is therefore vital to identify and assess mental health among the SUD population, as the co-occurrence of SUD and mental health disorders are likely to be underserved by current mental health systems [25, 26].

The aims of this prospective cohort study was to examine prevalence and change over time of mental health symptoms using the ten-item Hopkins Symptom Checklist (SCL-10) among people with severe substance use disorders (SUD) in Norway. In addition, the study aimed to assess potential predictors of mental health symptoms and change in symptom burden over time from substance use patterns and injecting use while also adjusting for level of education, living conditions, age and gender.

## Methods

### Study design and setting

This study is a nested prospective cohort study linked to the multicenter INTRO-HCV study [27]. The data was collected from May 2017 until July 2020 as part of an annual health assessment among people with SUD in nine OAT outpatient clinics in Bergen and Stavanger and two low-threshold municipality clinics in Bergen. The OAT clinics have implemented an integrated treatment and care model where patients are followed-up on a near daily basis by general and specialized nurses, psychologists and physicians who are under specialization- or specialized in addiction medicine. Buprenorphine-based and methadone are the two main OAT medications [28]. People with SUD in the municipality clinics are followed-up by social workers, general nurses and physicians specialized in family medicine. The INTRO-HCV study have employed trained research nurses who collected and completed the structured patient interviews, which were recorded in a health register using an electronic data collection software (CheckWare).

### Study sample

The study sample was comprised of two groups of patients; individuals diagnosed with opioid dependence (F11.2) according to World Health Organization’s International Classification of Diseases version 10 (ICD-10) [29], which were enrolled in OAT during the study period and accounted for 83% of the total study sample at baseline. The other participants were recruited from low-threshold municipality clinics among people who inject drugs. For the purpose of this paper, a SUD was defined as harmful use of, or dependency of a substance, and a *severe SUD* was defined as dependency of one or more substances. All included individuals were 18 years or older at time of inclusion and signed a written informed consent to partake in the study. Altogether 1042 SCL-10 measurements were included from 707 participants. Of the 707 participants with SCL-10 measures at baseline and 268 (38%) were included in a follow-up assessment with 67 (10%) having at least three annual measuring points. The mean time between SCL-10 measurements was 364 days (standard deviation (SD) 133). Table 1 shows details on clinical and sociodemographic characteristics of the study sample.

**Table 1:** Basic characteristics of study sample

Participants, n (%)	Baseline (n = 707)	Follow-up (n = 268)
**Gender**		
Male	500 (71)	208 (78)
Female	207 (29)	60 (22)
**Age, n (%)**		
18–29	83 (12)	25 (9)
30–39	203 (29)	71 (26)
40–49	217 (31)	87 (32)
50–59	161 (23)	71 (26)
≥ 60	43 (6)	14 (5)
Mean (SD)	43 (11)	45 (10)
**Highest education completed, n (%)**		
Not completed lower secondary school	41 (6)	15 (6)
Completed lower secondary school (9 years)	309 (44)	128 (48)
Completed upper secondary school (12 years)	285 (40)	99 (37)
Completed under or postgraduate studies (≥ 12 years)	72 (10)	26 (10)
**Current living conditions, n (%)**		
Stable (owned, rented or incarcerated)	619 (88)	242 (90)
Unstable (homeless, with family/friends)	88 (12)	26 (10)
**Worrying debt situation**	292 (41)	116 (43)
**Participants enrolled in OAT, n (%)**	590 (83)	248 (93)
OAT medications of those; n (%)		
- Methadone	224 (38)	110 (44)
- Buprenorphine-based	357 (61)	134 (54)
OAT treatment ratio*, mean (SD)	0.9 (0.4)	0.9 (0.3)
**Injecting and frequent substance use past 12 months, n (%)**		
Injected at least once	352 (54)	142 (53)
Alcohol	165 (25)	67 (25)
Cannabis	329 (50)	145 (55)
Stimulants (amphetamine/methamphetamine/cocaine)	183 (28)	73 (27)
Opioids (other than OAT)	103 (16)	29 (11)
Benzodiazepines	248 (38)	104 (39)

SD = standard deviation, OAT = Opioid agonist therapy,

*OAT ratio = ratio between daily OAT medication dose divided by expected mean daily dose; for buprenorphine 18 mg, buprenorphine-naloxone 18/4.5 mg or methadone 90 mg

Frequent substance use was defined as using substance at least weekly during the past 12 months

### Assessment

Measuring mental health status: Hopkins symptom check list (SCL-10).

The SCL-10 is a structured and self-administrated questionnaire, designed to measure symptoms of mental health disorders and psychological distress, and is widely used for both clinical and epidemiological purposes [30–32]. The SCL-10 involves ten items (suddenly scared for no reason, feeling fearful, faintness, dizziness or weakness, feeling tense, blaming yourself, difficulties falling asleep, feeling of worthlessness, feeling blue, feeling hopeless, and feeling everything is an effort), which are each scored on four dimensions from *not bothered at all* (item score = 1) to *extremely bothered* (item score = 4). Scores were summed and divided by the number of items answered to derive the mean item score. Mean scores vary between one and four, where the latter assumes *extremely bothered*. SCL-10 mean item scores were used for descriptive analyses while SCL-10 sum scores were used in linear mixed model (LMM) analyses. Furthermore, the mean item scores were calculated by gender, age, level of education, and living conditions at baseline. By introducing a cut-off point one can interpret the proportion of the respondents with symptoms of mental health disorders. A mean score of 1.85 for SCL-10 has been recommended as a threshold for indicating substantial mental health distress [31].

### Study variables; baseline, OAT, clinical and sociodemographic factors

Baseline was defined as the time when the first SCL-10 measure was completed upon the participant’s first annual health assessment. Subsequent SCL-10 measures at the next health assessment(s) were listed chronologically and included as follow-up. Being on OAT was defined as receiving either buprenorphine-based or methadone medication at baseline. Moreover, the OAT ratio, which corresponds to the received dose of OAT medication per day divided by expected mean dose (buprenorphine 18 mg or methadone 90 mg) according to World Health Organization [33], was calculated per OAT patient. For the clinical factors we defined *injecting substances* as having injected any substance during the last 12 months, and *frequent substance use* as using a substance more than once weekly during the last 12 months according to the subcategories of *alcohol*, *cannabis*, *stimulants* (amphetamine/methamphetamine/cocaine), *opioids* (non-OAT), and *benzodiazepines* (including z-hypnotics).

### Statistical analysis

All descriptive analyses were performed using STATA/SE 16.0. Expectation-maximization (EM) imputation and LMM analyses were performed in IBM SPSS version 26.0. Statistical significance was set at the *p* < 0.05 level. Missing values of SCL-10, clinical and sociodemographic variables, which included substance use, injecting substance use, educational level, worrying debt situation, and living conditions were assumed to be *missing at random* when performing EM imputation. There were missing values for 3.4% of these values, which were subsequently replaced with the estimated values by EM imputation according to Enders (2010) [34].

A LMM analyses were used to evaluate the impact of clinical and sociodemographic factors on the SCL-10 sum score. Time was defined as years from baseline Firstly, we ran a LMM analysis where each defined predictor variable was set against time, to assess whether the predictor variable changed over time. There were no clinical significant changes in these variables when analyzed separately as outcome variables – with the time variable being the exposure variable (data not shown). Thus, these predictor variables were included as constant and time-independent variables in further analyses. Secondly, a new LMM analysis was generated where these time-independent predictor variables were set against the SCL-10 sum score being the outcome variable. In addition, we added a time interactional to each predictor variable to investigate if time impacted changes of SCL-10 given each predictor. The predictor variables, on the baseline level and change in SCL-10 sum score, represented as main effects and interaction effects with time. The model was a random intercept fixed slope model with restricted maximum likelihood set as the estimator. This model uses all available data in the outcome variable.

## Results

### Basic characteristics of the study sample

Seventy-one percent of the study sample were male, mean (SD) age of 43 (11) at baseline and 45 (10) at follow-up for the whole cohort (Table 1). Approximately 40% had completed upper secondary school. Most participants (88%) had a stable living condition and 41% had a concerning debt situation. Eighty-two percent of the study sample was in OAT, of which 61 and 38% received buprenorphine-based medication and methadone, respectively. Over half had injected substances at least once during the last year, while 71% reported frequent substance use; most prevalent substances being cannabis (50%) and benzodiazepines (38%).

### SCL-10 scores at baseline and follow-up

**Fig. 1:**
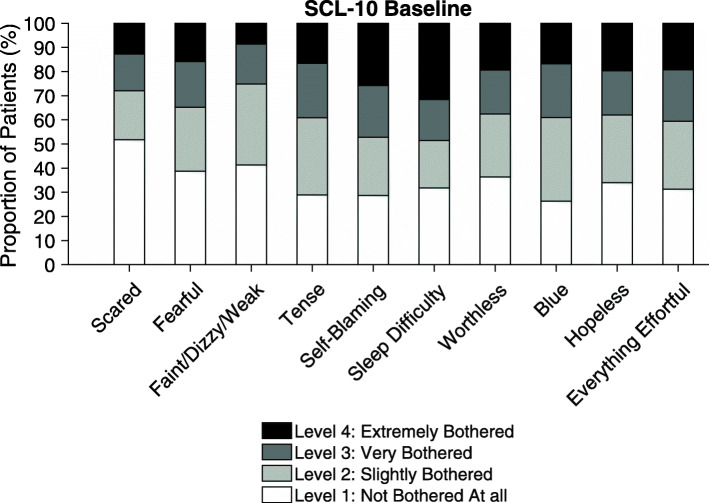
Proportion of SCL-10 item scores at baseline. SCL-10 = Symptoms checklist 10; ten items scale for measuring mental health status/psychological distress. The figure displays the proportion of patients responses on the ten item scale, from not bothered at all (item score= 1) to extremely bothered (item score = 4)

The mean (SD) of the SCL-10 item scores was 2.2 (0.8) (Table 2) at baseline. The distribution was sharply-peaked (kurtosis: 2.2) and slightly right-skewed (skewness: 0.4). The lowest mean (SD) item score (SD) was found for *suddenly scared for no reason* at 1.9 (1.1) and the highest score 2.5 (1.2) for *difficulty in falling asleep* (Fig. 1 and Additional File 1). Overall, females reported mean (SD) SCL-10 item score of 2.3 (0.8) and men 2.2 (0.8) [31].. People with unstable living conditions reported more symptoms of mental disorders than people with stable living conditions. Among OAT treatment, people on methadone reported mean (SD) SCL-10 of 2.3 (0.7) and buprenorphine-based medications at 2.2 (0.8).

**Table 2:** Baseline SCL-10 mean item scores and standard deviation (SD) by gender, age and sociodemographic factors

Baseline n = 707	SCL-10	
	Mean	SD
**Total**	**2.22**	**0.76**
Gender, n 707		
Male	2.17	0.76
Female	2.32	0.75
Age, n 707		
18-29	2.31	0.78
30-39	2.20	0.75
40-49	2.25	0.79
50-59	2.16	0.72
≥60	2.14	0.73
Highest level of education, n 705		
Not completed lower secondary school	2.46	0.78
Completed lower secondary school (9 years)	2.24	0.78
Completed upper secondary school (12 years)	2.14	0.72
Completed undergraduate studies (≤ 15 years)	2.28	0.77
Completed postgraduate studies (≥ 15 years)	2.16	0.66
Current living conditions, n 705		
Stable (owned, rented or incarcerated)	2.19	0.74
Unstable (homeless, with family/friends)	2.40	0.84
Enrolled in OAT and by medication, n 583		
Methadone	2.28	0.71
Buprenorphine	2.15	0.77

SCL-10 = Symptoms checklist 10; ten items scale for measuring mental health status/psychological distress, SD =standard deviation, OAT = opioid agonist therapy

SCL-10 = Symptoms checklist 10; ten items scale for measuring mental health status/psychological distress.

The figure displays the proportion of patients responses on the ten item scale, from *not bothered at all* (item score = 1) to *extremely bothered* (item score = 4).

We found vast individual dissimilarities in subjective mental health symptoms at baseline (Additional File 2); minimum and maximum mean SCL-10 item score was one and four, respectively. Thirty-three participants (4.7%) reported a mean of one; meaning *not bothered at all* on any items, while three participants (0.4%) were *extremely bothered* on all items. Sixty-five percent of the cohort reported a mean SCL-10 above the 1.85 cut-off point, which is recommended as a predictor of mental disorder [31] as shown in the Pen’s Parade below.

Pen’s Parade: SCL-10 = Symptoms checklist 10; ten items scale for measuring mental health status/psychological distress.

The figure displays distribution in SCL-10 mean values at baseline (*n* = 707) and follow up (*n* = 268), represented by fixed black line and vertical grey lines. The dotted lines represent the mean reported SCL-10 score of the Norwegian reference population (1.36) and standard reference of 1.85 indicating one or more mental disorders above this cut-off, respectively. Source: *Strand BH, Dalgard OS, Tambs K, Rognerud M: Measuring the mental health status of the Norwegian population: a comparison of the instruments SCL-25, SCL-10, SCL-5 and MHI-5 (SF-36). Nordic journal of psychiatry 2003* [31]*.*

**Fig. 2:**
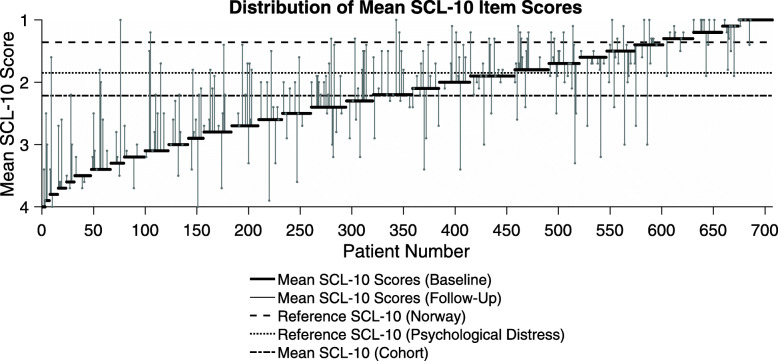
Pen’s Parade: Distribution of mean SCL-10 item scores at baseline and follow-up. Pen’s Parade: SCL-10 = Symptoms checklist 10; ten items scale for measuring mental health status/psychological distress. The figure displays distribution in SCL-10 mean values at baseline (n=707) and follow up (n=268), represented by fixed black line and vertical grey lines. The dotted lines represent the mean reported SCL-10 score of the Norwegian reference population (1.36) and standard reference of 1.85 indicating one or more mental disorders above this cut-off, respectively. Source: Strand BH, Dalgard OS, Tambs K, Rognerud M: Measuring the mental health status of the Norwegian population: a comparison of the instruments SCL-25, SCL-10, SCL-5 and MHI-5 (SF-36). Nordic journal of psychiatry 2003 [31]

Altogether 268 (38%) of the 707 participants at baseline had SCL-10 measures at two data points. As shown in Fig. 2, individual SCL-10 score at first follow-up are indicated with grey points and individual changes from baseline with vertical lines. Sharp changes go in both positive and negative directions and appear considerable for some.

### Impact of substance use patterns, clinical and sociodemographic factors on baseline level and change in SCL-10 sum score

Using a LMM analysis, we found higher SCL-10 sum scores at baseline for females (SCL-10 sum score: 1.8, 95% confidence interval (CI): 0.7 to 3.0) compared to men, people with unstable living conditions (1.7, CI: 0.0 to 3.3) and having a worrying debt (2.2, CI: 1.1 to 3.3) compared to people with stable living conditions and non-worrying debt, respectively. For substances, frequent use of cannabis (1.3, CI: 0.2 to 2.5), other opioids (2.7, CI: 1.1 to 4.2) and benzodiazepines (3.6, CI: 2.4 to 4.8) were associated with higher SCL-10 scores at baseline compared to people with no or non-frequent use of these substances (Table 3). On the other hand, frequent use of stimulants was associated with lower SCL-10 sum score at baseline (− 2.7, CI: − 4.1 to − 1.4) compared with people with no or less frequent use. There were no significant time interactions between any of the substance use patterns and changes in the SCL-10 sum score, nor were there any significant time interactions with sociodemographic characteristics.

**Table 3:** Linear mixed model of SCL-10 adjusted for clinical and sociodemographic factors

	Fixed effects	
	Baseline	Change per year
n = 707	Estimate (95% CI)	Slope (95% CI)
Factor impact* on SCL-10 sum score at baseline and changes per year from baseline		
SCL-10 sum score	18.1 (15.9 to 20.2)	0.6 (−1.6 to 2.9)
Female	*1.8 (0.7 to 3.0)*	0.4 (−0.9 to 1.8)
Age per 10 years	0.0 (−0.1 to 0.0)	0.0 (0.0 to 0.1)
**Clinical factors**		
Injecting substance use		
Injecting at least once last 12 months	0.6 (−0.7 to 1.8)	−0.3 (−1.6 to 1.0)
**Frequent use of substances**		
Alcohol	0.7 (−0.6 to 1.9)	0.1 (−1.2 to 1.4)
Cannabis	*1.3 (0.2 to 2.5)*	0.3 (−0.9 to 1.4)
Stimulants (amphetamines/ cocaine)	*−2.7 (−4.1 to − 1.4)*	− 0.2 (−1.6 to 1.3)
Opioids (other than opioid dispensed on OAT)	*2.7 (1.1 to 4.2)*	−2.6 (− 4.7 to − 0.4)
Benzodiazepines	*3.6 (2.4 to 4.8)*	− 0.4 (− 1.7 to 0.8)
**Sociodemographic factors**		
Level of education	−0.1 (− 0.7 to 0.6)	−0.6 (− 1.3 to 0.1)
Unstable living conditions	*1.7 (0.0 to 3.3)*	1.1 (−1.0 to 3.3)
Worrying debt situation	*2.2 (1.1 to 3.3)*	0.4 (−0.7 to 1.6)

SCL-10 = Symptoms checklist 10; ten items scale for measuring mental health status/psychological distress, CI = confidence interval

*Age per 10 years (centered according to mean age 43 years), level of education was coded 0–4 with 4 as the highest educational level, living conditions; unstable situation homeless or non-permanent residence, worrying debt situation: including any legal or illegal fees and debt, injecting substance use: during last 12 months

## Discussion

In this study, we found that 65% of people with SUD have symptoms of mental health disorders and psychological distress. Mental health symptoms were particularly prevalent among females, people with frequent use of cannabis, non-OAT opioids, and benzodiazepines compared to men and people with no or less frequent use of these substances. Interestingly, there were no clear associations between substance use patterns and change in mental health symptoms over time. This could suggest that the differences observed were indicating self-medication to larger degree than medication-related decline in mental health.

People with SUD are a heterogeneous population; fifteen and 35 % reported lower mean SCL-10 item scores compared to the general population and the standard reference score for symptoms of mental health disorders, respectively. Despite vast intra-individual variations in SCL-10 score from baseline to first follow-up, going in both directions, there were no time trends indicating change over time for the total study sample. This indicates that mental health disorders and psychological distress persist over time for this group and we are not able to explain the huge shift, positive and negative, in mental status of many individuals.

The mean SCL-10 for our cohort was 2.2, which is considerable lower compared to the general Norwegian population at 1.4, estimated to be around 11% of the population [31]. Around two-thirds of the total study sample reported symptoms of mental health disorders. This was somewhat higher symptom burden compared to cohort among people with SUD in Sweden [35], however, lower compared to a study among people entering SUD treatment in Norway, which found that over 80% had a level of mental distress above the 1.85 cut-off for SCL-10 at admission [36]. This could reflect that initiating SUD treatment, often combined with strict detoxification, is a very stressful event, whereas most of the patients included in our cohort were long-term OAT patients with a mean treatment time of almost eight years [13]. Correspondingly, follow-up studies have shown that there may be a significant reduction in SCL-10 symptoms when these individuals are discharged from inpatient treatment, however, presence of mental health disorders and severity of substance use seem to be independent predictors of considerable symptoms of mental health disorders in the long-term [37, 38]. We found that mental health symptoms at baseline were associated with a worrying debt situation, unstable living conditions and a frequent use of some of the substances. Severe debt has been found to correlate with poor mental health in a systematic review summarizing a number of studies [39]. There are also several studies suggesting a strong relationships between substance use and psychological distress, despite hardship to establish exact causality [40–42]. In the above study among people entering SUD treatment, severity of substance use, although stratified into alcohol use, illicit drug use and number of substances used– but not the actual substances used; was the most significant predictor of symptoms of mental health disorders [36]. However, again the question arises whether these symptoms are the direct result of the substance use or symptoms of mental distress presenting upon treatment admission [36].

In our study, use of cannabis, non-OAT opioids and benzodiazepines were co-occurring with mental health distress at baseline, while the opposite was seen for stimulants. There were no changes in time trends between use of substances and mental health symptoms. One hypothesis for these findings could be that the associations at baseline might be due to reverse causality, i.e. that participants with substantial mental health symptoms use substances to self-medicate symptoms [43]. It is also possible that there is a “flattening effect” and that potential negative impact of substances are more substantial at an earlier phase and that the change in later phases are less pronounced. Other research indicate that high doses of benzodiazepines reduce social functioning, and that it may also increase psychological distress and worsen mental health [16, 44], and misuse of benzodiazepines is seen among both SUD and psychiatric populations alike [45]. Similarly, the use of stimulants, in particular methamphetamine, has been associated with poor mental health outcomes [23]. Self-medication of attention deficit hyperactivity disorder (ADHD) with stimulants could be one explanation for these findings. Yet one study found that high ADHD symptom burden was associated with higher mental distress and use of simulants among OAT patients [46]. It is estimated that up to a third of patients in OAT have ADHD and previously we have found that coverage of central acting stimulants in this patient group is very low [12, 47, 48]. An alternative explanation could be that stimulants have a direct positive impact on mental health symptoms among these patients. However, the time trend analyses does not support these hypotheses.

Although prevalence of mental disorders and SUD comorbidity has been found to vary among European countries; research consistently shows a high total prevalence of around 50%, with depression, anxiety disorders and personality disorders being the most frequent [9]. However, some facility based studies indicate an even higher comorbidity prevalence as people with severe symptoms are more likely to seek support; 70% for personality disorders [3] and a lifetime substance-independent mental disorder was found in nine out of ten patients enrolled in treatment facilities [49]. Comorbid mental health disorders and SUD have been found to be associated with poor treatment outcomes and show a higher psychopathological severity compared to people with a single disorder [50–52], and this underlies the importance of assessing mental health status in clinical settings among people with SUD. We endorse that evaluation of mental health and linkage to mental health care services should be included in OAT programs and low-threshold SUD clinics; be gender-sensitive and follow and integrated treatment approach, which have been found superior compared to separate treatment plans [53–55].

The major strength of this study is the relatively large sample size of a “hard-to-reach” population of people with SUD as well as a cohort design. However, there are some limitations. Firstly, only a minority contributed to the prospective analyses (268/707). To reduce the potential for selection bias between the sub-group with follow-up SCL-10 measurements presented in Fig. 2 and the baseline cohort, we conducted an inverse probability weighted analysis. Our study sample is also mainly relevant for people with opioid dependence being enrolled in OAT treatment as most were in this group. Thus, our research might not be generalized to other groups with SUD. Moreover, both in the OAT and low-threshold SUD clinics, patient- and system delays contributed to non-accurate annual health assessments, which could in turn affect both answers and results. Thirdly, the SCL-10 has limitations. It is not a diagnostic tool for mental health disorders and is no replacement for clinical interviews and more comprehensive psychiatric instruments among people with SUD. Literature also suggests that the SCL-10 predicts depression and anxiety better than other diagnosis, and that some 50–60% of the patients identified with symptoms of mental disorders qualify for at least one or more mental disorders when assessed clinically [31, 56, 57].

## Conclusion

People with SUD have considerable symptoms of mental health disorders and psychological distress. However, this is a diverse and dynamic population with extreme individual variations. Around one-third have few symptoms of mental health disorders. This emphasizes the importance of consideration and evaluation of symptoms of mental health disorders and psychological distress in both OAT and low-threshold SUD clinics to further improve personalized patient care. Mental health problems were particularly observed among females, people with frequent use of cannabis, opioids, and benzodiazepines, and less among people using amphetamines. Time trend analyses could suggest that the differences observed indicates self-medication or a flattening effect rather than medication-related decline in mental health. Studies with long term follow-up or experimental design is needed to confirm these potential effects better.

## Supplementary Information

**Additional file 1.** SCL-10 = Symptoms checklist 10; ten items scale for measuring mental health status/psychological distress, SD = standard deviation,

**Additional file 2. **Pen’s Parade: SCL-10 = Symptoms checklist 10; ten items scale for measuring mental health status/psychological distress. The figure shows distribution in SCL-10 mean values at baseline (*n* = 707) by fixed black line. The dotted lines represent the mean reported SCL-10 score of the Norwegian reference population (1.36) and standard reference of 1.85 indicating one or more mental disorders above this cut-off, respectively. Source: *Strand BH, Dalgard OS, Tambs K, Rognerud M: Measuring the mental health status of the Norwegian population: a comparison of the instruments SCL-25, SCL-10, SCL-5 and MHI-5 (SF-36). Nordic journal of psychiatry 2003.*

## Data Availability

Dataset used for SCL-10 for this publication may be available in an anonymous and shortened version upon contacting the corresponding author.

## Abbreviations

ADHD: Attention deficit hyperactivity disorder
EM: Expectation-maximization imputation
INTRO-HCV: Integrated treatment of hepatitis C virus infection
HCV: Hepatitis C virus infection
LMM: Linear mixed model
OAT: Opioid agonist therapy
SCL-10: Hopkins Symptom Check List 10
SCL-25: Hopkins Symptom Check List 25
SUD: Substance use disorder
