# Supplementary figures and images for: Substance use and symptoms of mental health disorders: a prospective cohort of patients with severe substance use disorders in Norway
Source: Subst Abuse Treat Prev Policy. 2021 Feb 27;16:20. doi: 10.1186/s13011-021-00354-1 (PMC7912462; doi:10.1186/s13011-021-00354-1)

# Distribution of Mean SCL-10 Item Scores

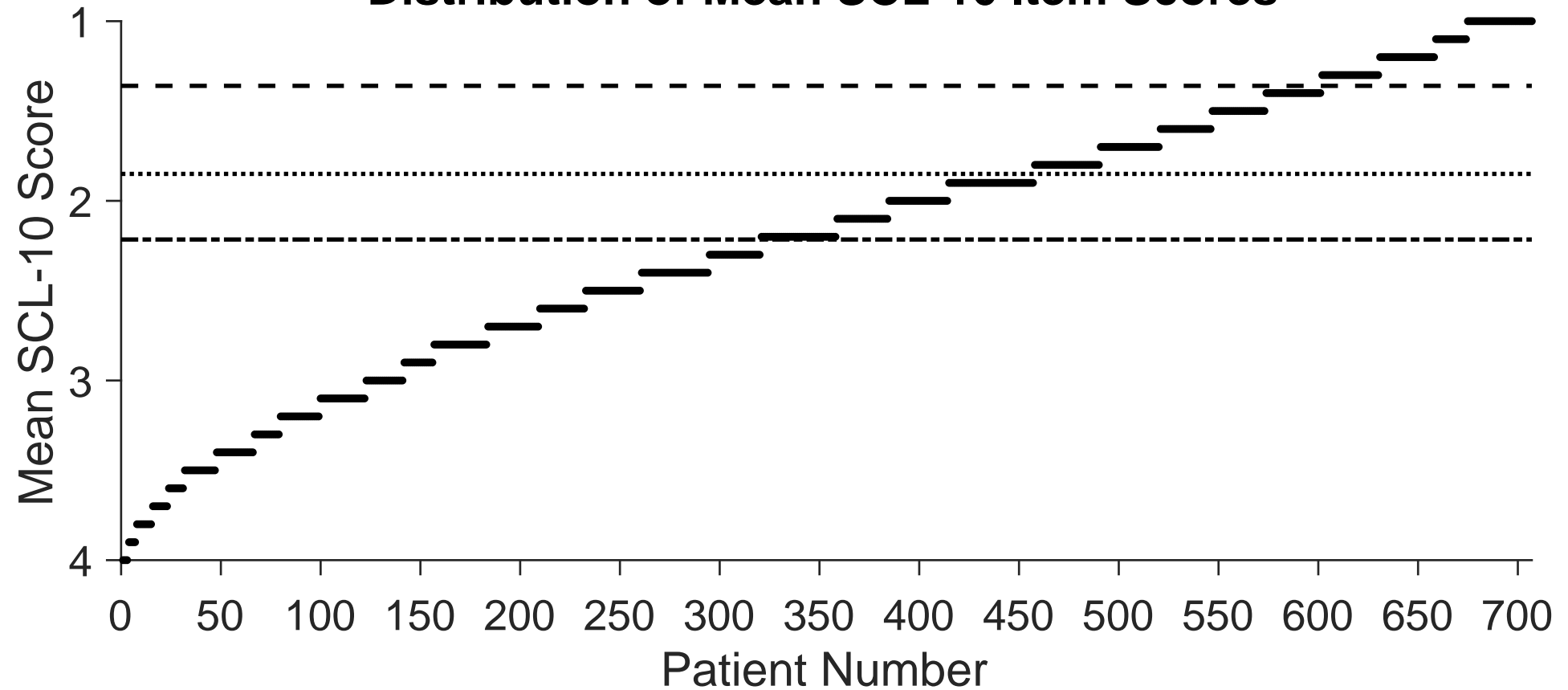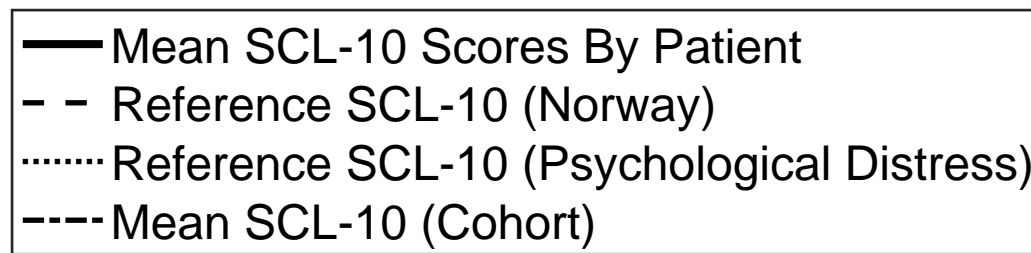

Supplement: Supplementary file 2 — Additional file 2. Pen’s Parade: SCL-10 = Symptoms checklist 10; ten items scale for measuring mental health status/psychological distress. The figure shows distribution in SCL-10 mean values at baseline (n = 707) by fixed black line. The dotted lines represent the mean reported SCL-10 score of the Norwegian reference population (1.36) and standard reference of 1.85 indicating one or more mental disorders above this cut-off, respectively. Source: Strand BH, Dalgard OS, Tambs K, Rognerud M: Measuring the mental health status of the Norwegian population: a comparison of the instruments SCL-25, SCL-10, SCL-5 and MHI-5 (SF-36). Nordic journal of psychiatry 2003. [file 13011_2021_354_MOESM2_ESM.pdf]
